# High-sensitivity cardiac troponin T is associated with disease activity in patients with inflammatory arthritis

**DOI:** 10.1371/journal.pone.0281155

**Published:** 2023-02-10

**Authors:** Thao H. P. Nguyen, Morten Wang Fagerland, Ivana Hollan, Jon Elling Whist, Mark W. Feinberg, Stefan Agewall

**Affiliations:** 1 Lillehammer Hospital for Rheumatic Diseases, Lillehammer, Norway; 2 Faculty of Medicine, Institute of Clinical Medicine, University of Oslo, Oslo, Norway; 3 Department of Rheumatology, Oslo University Hospital, Rikshospitalet, Oslo, Norway; 4 Oslo Centre for Biostatistics and Epidemiology, Research Support Services, Oslo University Hospital, Oslo, Norway; 5 Beitostølen Health and Sport Centre, Beitostølen, Norway; 6 Norwegian University of Science and Technology, Gjøvik, Norway; 7 Department of Laboratory medicine, Innlandet Hospital Trust, Lillehammer, Norway; 8 Harvard Medical School, Boston, MA, United States of America; 9 Division of Cardiology, Brigham and Women´s Hospital, Boston, MA, United States of America; 10 Department of Cardiology, Oslo University Hospital, Ullevål, Oslo, Norway; University of Toronto, CANADA

## Abstract

**Objective:**

To investigate whether high-sensitivity cardiac troponin T (hsTnT) correlates to markers of disease activity in inflammatory arthritis (IA), and whether antirheumatic treatment influences hsTnT levels.

**Methods:**

We assessed 115 patients with active IA (64 rheumatoid arthritis (RA), 31 psoriatic arthritis and 20 ankylosing spondylitis) before and after using methotrexate (MTX) alone or tumor necrosis factor inhibitor (TNFi) with or without MTX co-medication (TNFi±MTX). All patients starting with TNFi had been previously unsuccessfully treated with MTX monotherapy. HsTnT (measured in serum by electro-chemiluminescence immunoassay (Roche Elecsys® Troponin T- high-sensitivity)), and other clinical and laboratory parameters were evaluated at baseline, and after 6 weeks and 6 months of treatment.

**Results:**

Of markers of disease activity, baseline levels of hsTnT positively correlated with Physicians’ Global Assessment Score of disease activity in the total patient cohort (p = 0.039). In RA group, hsTnT positively correlated with swollen joints, Disease Activity Score for 28 joints with ESR and serum tumor necrosis factor levels (p = 0.025, p = 0.008, p = 0.01, respectively). Median hsTnT at baseline was 5.0 ng/L, and did not change significantly at 6-week visit (6.0 ng/L, p = 0.37) and 6-month visit (6.0 ng/L, p = 0.18) with either antirheumatic therapy.

**Conclusions:**

HsTnT levels were associated with inflammatory markers for IA disease activity. However, while inflammatory markers significantly improved after antirheumatic treatment, hsTnT did not change during the 6-month follow-up period.

## Introduction

Compared to the general population, patients with inflammatory arthritis (IA) suffer a substantial risk of developing coronary heart disease (CHD), independently of conventional cardiovascular risk factors [1].

High-sensitivity cardiac troponin T (hsTnT), a marker of myocyte necrosis and injury, has emerged as a strong predictor of cardiovascular disease (CVD) or mortality in at-risk populations, even in early stages of disease [2, 3].

HsTnT has been observed to be increased in IA patients [4–6]. The mechanisms by which cardiac troponins are elevated in IA patients are unclear, but accumulating evidence suggests the contributing role of systemic inflammation and the instability and rupture of atherosclerotic plaques [7, 8]. HsTnT does not only reflect acute coronary syndrome, but also chronic atherosclerotic disease and localized myocardial necrosis, and correlates with coronary plaque burden [9, 10]. In individuals without clinically manifest myocardial damage or previous CHD, hsTnT concentration within the normal range is associated with structural heart disease [11]. Early identification of high-risk patients would potentially allow for precision medicine approach for intervention of IA comorbidity.

Based on the known, increased CHD risk in IA patients, and possible link between myocardial injury and inflammation, we hypothesized that hsTnT levels would correlate with systemic inflammatory and clinical markers for IA disease activity, and may be influenced by antirheumatic therapy.

## Materials and methods

### Participants

This study is a part of PSARA—the Norwegian prospective observational Psoriatic arthritis (PsA), Ankylosing spondylitis (AS), Rheumatoid Arthritis (RA) study, described earlier [12, 13]. We enrolled 115 patients with active IA starting with either methotrexate (MTX) monotherapy or tumor necrosis factor inhibitor (TNFi) with or without MTX co-medication (TNFi±MTX). Patients met criteria for PsA by the modified Caspar criteria for PsA [14], for AS by the modified New York diagnostic criteria for AS [15], or for RA by the American Rheumatism Association 1987 revised classification criteria for RA [16]. Medicines were administrated in common recommended doses according to clinical judgment performed by a rheumatologist not involved in this study, in accordance with the Norwegian guidelines that adhere to the main international recommendations [17–19]. All patients starting with MTX were MTX naïve, and all recipients of TNFi were also TNFi naïve, and had been previously unsuccessfully treated with MTX, except for AS group wherein TNFi was the treatment of choice in patients with persistently high disease activity despite nonsteroidal anti-inflammatory drugs [18].

The protocol was approved by Regional Committee for Medical and Health Research Ethics in Norway (s-07377b). All individuals were Caucasians and gave written informed consent.

### Clinical and laboratory tests

Patients were examined at baseline and after 6 weeks and 6 months of therapy. Data collection included demographic, clinical and therapeutic data, life-style parameters and self-reported questionnaires.

Venous blood samples were drawn after fasting of eight hours. Routine hematologic and biochemical tests were consecutively performed at the local hospital laboratory. Additional blood samples for later analyses (including hsTnT) were immediately prepared, divided into small aliquots, and stored at -80°C until analyzed.

HsTnT was measured in serum by electro-chemiluminescence immunoassay (Roche Elecsys® Troponin T- high-sensitivity). Level of 5 ng/L is the limit of detection of this assay and was used as the cut‐off for our detectable hsTnT category. A concentration of 14 ng/L or higher represents the 99th percentile value for a healthy reference population, and is referred to as elevated [20]. Gender-specific 99th percentiles for females and males are 13.1 ng/L and 16.8 ng/L, respectively [21]. The threshold for elevated hsTnT was established primarily from populations of healthy persons without CVD [20, 21]. Former studies have found that approximately 2% of adults (and 4.6% of those aged 50 to 60 years) without symptoms of acute ischemia have a hsTnT level higher than the threshold [22, 23].

### Statistical analysis

Continuous data were expressed as median (interquartile range) and categorical data by number (proportion) as appropriate. Chi-squared tests were used for comparisons of categorical data, and independent samples t-tests, paired t-tests and one-way ANOVA were used for comparisons of continuous data between groups. In order to maintain the nominal probability of a type I error when making multiple statistical tests, we used the Bonferroni correction in comparison of data between subgroups. Spearman’s rank-order coefficients, with 95% confidence intervals based on the Fisher Z transformation, were calculated to evaluate correlations between hsTnT, IA and CHD related variables at all points of time. The multiple regression models were adjusted for age and gender and for the baseline characteristics that were statistically significantly related to hsTnT in simple regression analysis.

A two-tailed probability value of p < 0.05 was considered statistically significant. All statistical analyses were carried out with Stata/SE 16 (StataCorp LLC, College Station, TX).

## Results

### Baseline characteristics of patients

The baseline characteristics are presented in Table 1.

**Table 1:** Baseline characteristics.

	All patients	RA	PsA	AS	MTX	TNFi±MTX
	(n = 115)	(n = 64)	(n = 31)	(n = 20)	(n = 51)	(n = 64)
HsTnT (ng/L)	5.0(3.5-7.0)	5.0 (2.6-7.0)	6.0 (5.0-8.0)	6.0 (3.0-8.0)	5.0 (3.5-7.0)	6.0 (3.3-7.0)
Age (years)	56 (47-62)	**57.5(51.5-63)^ϕ^^φ^**	**50 (43-61)^ϕ^**	**49 (43.5-59)^φ^**	56 (49-63)	55 (46-61)
Gender, women, n (%)	64 (55.7)	**47 (73.4)^ϕ^^φ^**	**13 (41.9)^ϕ^**	**4 (20.0)^φ^**	31 (60.8)	33 (51.6)
Disease duration (years)	2.0 (0.1-10)	1.5 (0.1-8)	2.0 (0.2-13)	2.5 (1.0-6.5)	**0.1 (0.1-3.0)** *	**3.8(1.5-12.5)** *
**Treatment, n (%)**						
MTX monotherapy	51 (44.4)	34 (53.1)	17 (54.9)	0	51 (100)	0
TNFi ± MTX	64 (55.7)	30 (46.9)	14 (45.1)	20 (100)	0	64 (100)
**Disease activity**						
CRP (mg/L)	7.0 (3.0-14)	8.0 (3.0-16)	5.0 (2.0-10)	7.5 (3.0-14.5)	8.0 (3.0-19)	6.5 (3.0-12.5)
ESR (mm/h)	13.0 (6-26)	**18.5 (8-29.5)**	**7.5 (4-16)^ϕ^**	9.5 (7-16.5)	14.5 (7-30)	13.0 (5-24)
WBC (10⁹/L)	7.1 (5.9-8.6)	7.25 (5.9-8.6)	6.35(4.8-8.1)	7.9 (6.35-8.6)	6.9 (5.7-8.2)	7.3 (5.9-8.65)
RF IgM, n(%)	NA	45 (70.3)	NA	NA	22 (44.0)	23 (76.7)
ACPA, n(%)	NA	39 (60.9)	NA	NA	**17 (50.0)** *	**22 (73.3)** *
Erosive arthritis, n(%)	NA	34 (53.1)	NA	NA	**12 (35.3)** *	**22 (73.3)** *
Subcutanous nodules, n(%)	NA	12(18.8)	NA	NA	**3 (8.8)** *	**9 (30)** *
Swollen joinsts	NA	6 (3-9.5)	NA	NA	5.5 (3-10)	6 (3-9)
DAS28-ESR	NA	5.2 (4.3-5.7)	NA	NA	5.2 (4.6-5.7)	5.1 (4.2-5.7)
PGA	3.4 (2,3-4,7)	**3.9 (2.8-4.9)^ϕ^**	**2.3(1.6-4.1)^ϕ^**	3.0 (2.4-4.5)	3.6 (2.7-4.7)	3.1 (2.0-4.7)
PtGA	5.0 (3.1-6.7)	5.2 (3.8-6.7)	4.3 (2.6-5.9)	5.6 (3.1-7.2)	5.2 (3.2-6.4)	4.9 (3.0-7.1)
MHAQ	0.5 (0.3-0.8)	0.65 (0.28-0.9)	0.4 (0.3-0.7)	0.43(0.25-0.73)	0.45(0.3-0.75)	0.5(0.28-0.85)
BASDAI	NA	NA	**4.3 (3.0-5.9)°**	**5.6 (4.4-7.3)°**	4.5 (3.3-6.4)	4.9 (3.7-6.7)
**CVD risk factors**	** **	** **	** **	** **	** **	** **
Etabished CVD	14 (12.2)	8 (12.5)	**1 (3.2)°**	**5 (25.0)°**	4 (7.8)	10 (15.6)
Hypertention	30 (26.1)	17 (26.6)	7 (22.6)	6 (30.0)	9 (17.7)	21 (32.8)
BMI (kg/m^2^)	26.1(23.5-29.4)	26.1 (23.6-28.5)	26 (23-29)	27.9(24.2-31.4)	26(23.6-27.9)	27.2(23.3-31.8)
Hyperlipidemia	17 (14.8)	11 (17.2)	3 (9.7)	3 (15.0)	9 (17.7)	8 (12.5)
Current smokers	37 (32.2)	20 (31.3)	7 (22.6)	10 (50.0)	16 (31.4)	21 (32.8)
Diabetes	4 (3.5)	3 (4.7)	0	1 (5.0)	0	4 (6.3)
**Comedication,n (%)**						
NSAIDs	76 (66.1)	**47 (73.4)^ϕ^**	**15 (48.4)^ϕ^**	14 (70.0)	36 (70.6)	40 (62.5)
Glucocorticoids	22 (19.1)	**17 (26.6)^ϕ^^φ^**	**3 (9.7)^ϕ^**	**2 (10)^φ^**	8 (15.7)	14 (21.9)
Beta-blockers	10 (8.9)	**5 (7.8)^ϕ^^φ^**	**1 (3.5)^ϕ^**	**4 (20.0)^φ^**	4 (8.0)	6 (9.5)
CCB	9 (8.0)	5 (7.8)	2 (7.1)	2 (10.0)	2 (4.0)	7 (11.3)
Statins	20 (17.4)	**12 (18.8)^ϕ^**	**1 (3.2)^ϕ^°**	**7 (35.0)°**	7 (13.8)	13 (20.3)
Acetyl salicylic acid	11 (9.6)	6 (9.4)	2 (6.5)	3 (15)	6 (11.8)	5 (7.8)

All values are given as median (interquartile range), unless otherwise specified.

* p-value < 0.05 for comparison between MTX group and TNF group

^ϕ^ p-value < 0.05 for comparison between RA group and PsA group

^φ^ p-value < 0.05 for comparison between RA group and AS group

° p-value < 0.05 for comparison between AS group and PsA group

Established CVD defined as previous presence of any of these conditions: Angina pectoris, stroke, myocardial infarction, carotid stenosis, chronic heart failure, percutaneous coronary angioplasty, aortic aneurysm.

Abbreviations: RA: Rheumatoid arthritis, PsA: psoriatic arthritis, AS: ankylosing spondylitis, MTX: Methotrexate, TNFi: tumor necrosis factor inhibitors, hsTNT: high-sensitivity cardiac troponin T, CRP: C-reactive protein, ESR: erythrocyte sedimentation rate, WBC: white blood cells, RF-IgM: rheumatoid actor immunoglobulin M, ACPA: Anti-citrullinated protein antibody, DAS28-ESR: Disease Activity Score for 28 joints with ESR, PGA: Physician’s Global Assessment Score of disease activity, PtGA: Patient’s Global Assessment Score of disease activity, MHAQ: Modified Health Assessment Questionnaire, BASDAI: Bath Ankylosing Spondylitis Disease Activity Index, BMI: body mass index, NSAIDs: non-steroidal anti-inflammatory drugs, CCB: Calcium channel blockers, NA: not applicable.

Patients with RA were significantly older than those in the PsA and AS group (median 57.5 versus 50, p = 0.005 and 49 years old, p = 0.003, respectively). Disease activity was high in all the subgroups. AS patients had significantly higher frequency of established CVD, defined as angina, myocardial infarction, peripheral vascular disease and/or stroke. Baseline characteristics of RA patients were similar in both treatment groups, except for longer disease duration, higher incidence of erosive arthritis, subcutaneous nodules, and anti–citrullinated protein antibody positivity in the TNF±MTX group (Table 1).

### Associations between hsTnT and IA-related risk factors

Median baseline hsTnT was 5.0 ng/L [IQR 3.5–7.0], with no significant difference between all subgroups. A total of 7 patients (6.1%) had an hsTnT>14 ng/L which is the upper reference limit (99th percentile) for hsTnT when using Elecsys Troponin T assay; Roche.

In multiple regression analyses adjusted for age and gender, hsTnT positively associated with Physicians’ Global Assessment Score of disease activity (PGA) in the total patient cohort. In RA group, hsTnT positively associated with swollen joints, Disease Activity Score for 28 joints with ESR (DAS28-ESR) and tumor necrosis factor (TNF) (Table 2). However, association between hsTnT and DAS28-ESR and TNF was only observed in RA patients using TNFi+MTX (p = 0.050 and p = 0.005, respectively).

**Table 2:** Associations between hsTnT levels and selected variables at baseline.

	Unadjusted analyses			Adjusted analyses		
				Multiple linear regression		
	Spearman´s rho	95% CI	P-value	Coefficient	95% CI	P-value
**All patients**						
CRP (mg/L)	0.05	-0.14 to 0.23	0.60	0.002	-0.04 to 0.04	0.91
ESR (mm/h)	0.008	-0.18 to 0.19	0.93	0.02	-0.03 to 0.07	0.49
WBC (10⁹/L)	-0.06	-0.24 to 0.12	0.52	-0.09	-0.39 to 0.56	0.72
PGA	*0*.*17*	*-0*.*01 to 0*.*35*	*0*.*07*	**0.58**	**0.03 to 1.14**	**0.039**
PtGA	**-0.21**	**-0.38 to -0.03**	**0.022**	**-0.63**	**-1.03 to -0.24**	**0.002**
MHAQ	0.003	-0.18 to 0.19	0.98	-0.21	-2.65 to 2.24	0.87
Age	**0.29**	**0.05 to 0.50**	**0.002**	**0.13**	**0.05 to 0.21**	**0.001**
**RA group**	** **	** **	** **	** **	** **	** **
TNF (ng/L)	0.17	-0.09 to 0.40	0.19	**1.56**	**0.38 to 2.73**	**0.01**
Swollen joints	0.20	-0.04 to 0.43	0.11	**0.20**	**0.03 to 0.37**	**0.025**
DAS28-ESR	*0*.*24*	*-0*.*01 to 0*.*46*	*0*.*057*	**1.47**	**0.41 to 2.53**	**0.008**

Bold values denote statistical significance at the p < 0.05 level.

Abbreviations: CRP: C-reactive protein, ESR: erythrocyte sedimentation rate, WBC: white blood cells, TNF: tumor necrosis factor, DAS28-ESR: Disease Activity Score for 28 joints with ESR, PGA: Physician’s Global Assessment Score of disease activity, PtGA: Patient’s Global Assessment Score of disease activity, MHAQ: Modified Health Assessment Questionnaire.

HsTnT levels were negatively associated with Patients’ Global Assessment Score of disease activity (PtGA) in both simple regression analyses, and after adjustment for age and gender (Table 2).

Changes in hsTnT were positively associated with changes in swollen joints and TNF in all RA patients after 6 weeks on antirheumatic treatment (rho = 0.30 [95%CI = 0.06 to 0.51], p = 0.016 and rho = 0.26 [95%CI = 0.01 to 0.48], p = 0.041, respectively). However, we did not observe these associations at 6-month visit (rho = 0.12 [95%CI = -0.13 to 0.355], p = 0.37 and rho = 0.13 [95%CI = -0.12 to 0.37], p = 0.30, respectively).

No further significant relationships were noted, neither in simple nor in multiple linear regression analyses.

### Effects of antirheumatic treatment

Table 3 shows changes in hsTnT and selected markers related to IA during therapy. Both circulating and clinical inflammatory markers for IA disease activity, significantly improved with antirheumatic treatment. In contrast, serum levels of hsTnT did not seem to be influenced by antirheumatic treatment (p_baseline-6weeks_ = 0.37; p_baseline-6months_ = 0.18). No significant differences between diagnosis groups or between treatment groups were identified (Table 3).

**Table 3:** Changes in hsTnT and some selected clinical and laboratory variables.

	All (n = 115)			RA (n = 64)			PsA (n = 31)			AS (n = 20)			MTX (n = 51)			TNFi±MTX (n = 64)		
	B	6w	6m	B	6w	6m	B	6w	6m	B	6w	6m	B	6w	6m	B	6w	6m
hs-TnT (ng/L)	5.0	6.0	6.0	5.0	5.0	5.0	6.0	6.0	6.0	6.0	6.0	6.0	5.0	6.0	5.0	6.0	6.0	6.0
CRP (mg/L)	7.0	2.0***	2.0	8.0	2.0***	2.0	5.0	3.0**	2.0	7.5	1.0***	1.0	8.0	3.0***	2.0*	6.5	1.0***	2.0
ESR (mm/h)	###	8.0***	7.0	19	10.5***	9.5	7.5	6.5*	6.0	9.5	3.5***	3.0	15	12.0*	9.0**	13.0	5.5***	7.0*
WBC (10⁹/L)	7.1	6.3***	5.9	7.3	6.2***	5.9	6.4	5.9	5.5^ϕ^	7.9	6.7***	6.4	6.9	6.2**	5.8*	7.3	6.4***	6.3
TNF (ng/L)				1.2	0.97	1.0							1.2	0.96***	0.92	1.2	0.97	1.2
Swollen joints				6.0	2.0***	0.0***							5.5	3.5***	1.0***	6.0	1.0***	0.0*
DAS28-ESR				5.2	3.5***	2.8							5.2	4***	2.9*	5.1	3.1***	2.6
PGA	3.4	2.0***	1.4***	3.9	2.3***	1.4***	2.3	1.7***	1.3*	3.0	1.8***	1.1	3.6	2.4***	1.8*	3.1	1.8***	1.2***
PtGA	5.0	2.6***	1.6**	5.2	2.9***	1.5**	4.3	2.0***	2.0	5.6	3.0***	1.6	5.2	3.0***	2.0	4.9	2.5***	1.5*
MHAG	###	0.25***	0.20*	0.7	0.30***	0.20*	###	0.30**	0.25	0.4	0.23**	0.20	0.5	0.28***	0.15	0.50	0.25***	0.20
BASDAI							4.3	1.9***	2.3	5.6	2.2***	2.5	4.5	2.1*	2.3	4.9	1.9***	3.1

Bold values denote statistical significance at the p < 0.05 level.

* denotes statistically significant changes from baseline to 6 weeks and from 6 weeks to 6 months

* denotes significant changes with a p—value < 0.05

** denotes significant changes with a p—value = 0.001–0.01

*** denotes significant changes with a p—value < 0.001

^ϕ^ denotes statistically significant changes in WBC from baseline to 6 months

Abbreviations: B: At baseline, 6w: At 6-week visit, 6m: At 6-month visit, RA: Rheumatoid arthritis, PsA: psoriatic arthritis, AS: ankylosing spondylitis, MTX: Methotrexate, TNFi: tumor necrosis factor inhibitors, hsTNT: high-sensitivity cardiac troponin T, CRP: C-reactive protein, ESR: erythrocyte sedimentation rate, WBC: white blood cells, TNF: tumor necrosis factor, DAS28-ESR: Disease Activity Score for 28 joints with ESR, PGA: Physician’s Global Assessment Score of disease activity, PtGA: Patient’s Global Assessment Score of disease activity, MHAQ: Modified Health Assessment Questionnaire, BASDAI: Bath Ankylosing Spondylitis Disease Activity Index.

## Discussion

In accordance with previous studies [4, 5], we observed a positive correlation between myocardial injury in term of hsTnT and IA disease characteristics, measured by PGA for the total patient cohort, and by swollen joints and DAS28-ESR for RA patients, that remained valid after adjustment for age and gender. Similar association was identified between hsTnT and TNF.

Actually, DAS28-ESR was measured only in patient with RA. Therefore, we did not have data to explore the association between hsTnT levels and DAS28-ESR in the AS and PsA subgroups. We found no significant association between hsTnT levels and swollen joints in the AS and PsA subgroups. Only two of twenty AS patients and eighteen of thirty-one PsA patients in our population had swollen joints, while all of sixty-four RA patients had at least one swollen joint. Thus, the small sample size leads to low power for the subgroup analyses.

Observational studies have shown the correlation between the disease activity in arthritis and increased CVD risk, and suggest that the higher the inflammatory load, the more accelerated the processes of atherosclerosis become [24]. Furthermore, TNF, known as one of the main proinflammatory cytokines regulating the inflammatory response in autoimmune arthritis, is associated with the severity of subclinical atherosclerosis in RA, regardless of traditional cardiovascular risk factors [25, 26]. Hence, the relationship between markers of disease activity and cardiac biomarkers is of great interest. The information given by hsTnT, and TNF and DAS28-ESR in our study might be relevant in myonecrosis and inflammation that are thought to be involved in the pathogenesis of CHD. Thus, our observations might be related to the results of population-based studies, demonstrating a 30–50% increased risk of CHD in patients with IA [1]. Taken together, our findings may support the link between myocardial injury and systemic inflammation, and accentuate the importance of performing a thorough assessment for cardiovascular risk factors in IA patients.

Remarkably, no correlation between hsTnT and C-reactive protein, a routinely assessed marker of systemic inflammation in IA, was found. Lack of such a correlation was also noted in in previous studies conducted in patients with relatively well-controlled disease and also in patients with moderate to severe RA [27, 28]. This might suggest that active inflammation may not be the primary driver of troponin elevation in IA.

The impact of disease-modifying anti-rheumatic drugs on myocardial injury in term of hsTnT has been investigated in the literature, more extensively in RA as compared to AS and PsA. In contrast to markers for disease activity, and contrary to a previous study [27], initiation of MTX or MTX combined with an TNFi in RA and PsA patients and TNFi monotherapy in AS patients, did not lead to reduction in circulating levels of hsTnT during 6-month follow-up period. One may expect that reduction of inflammation in patients with IA would result in decreased levels of hsTnT, but a direct effect of antirheumatic therapy on hsTnT could not be ruled out in the present study.

The most likely explanation could be due to the low prevalence of myocardial injury in term of hsTnT (only 7 of 115 patients had increased hsTnT value compared to the reference healthy population), and the baseline levels of hsTnT were near the limit of detection of the high-sensitivity assay, which is 5 ng/L [20], also in patients with AS, although the occurrence of established CVD was significantly higher in AS group as compared to RA and PsA group. This might indicate a normal myocardial structure and function. Therefore, subsequently, the absolute change in hsTnT over time were small, and our study is not powered to detect such small changes. It is more unlikely to find a decrease in hsTnT in a group of patients with normal basal hsTnT levels.

Moreover, it is also possible that the duration of follow-up of 6 months was too short a time period to uncover significant changes on levels of hsTnT. We cannot exclude the possibility that hsTnT changes would become manifest if antirheumatic therapy occurred at later pre-symptomatic stages of myocardial injury at a time when hsTnT levels are known to be higher. In addition, atherosclerosis, a chronic inflammatory disease, typically results in episodic stages of plaque progression and quiescence. This study may not have been adequately captured atherosclerotic sequelae including low-level myocardial ischemic injury during this time period. The effect of antirheumatic treatment in patients with the highest quartile of hsTnT levels compared to the lowest would be of future interest to explore in larger studies.

Notably, we did not observe correlation between circulating levels of NT-proBNP and those of hsTnT. In our recently published study [12], we found a positive correlation between NT-proBNP and ESR and CRP, but similarly to hsTnT, antirheumatic treatment was not related to reduction in NT-proBNP levels during the 6-month follow-up study. A standard 12-lead ECG investigations were also performed in 91 patients (70.1% of the study population). Of those, the ECG was entirely normal in 84.6%.

An advantage of our study is the comprehensive characterization of patient population and the design that makes it feasible to compare the effect of the two antirheumatic treatment regimens on levels of hsTnT in IA. Weaknesses include the relatively small sample size, which may increase the risk of Type II-errors. Our study is also limited by its observational design, and lack of a healthy control group matched for age and sex with IA patients enrolled in the study. Results emerged from this type of study should be taken carefully. Prospective studies will be needed to evaluate the temporal relationship between disease activity and hsTnT.

In conclusion, hsTnT levels were associated with markers for IA disease activity, but did not improve during a 6-month period of antirheumatic treatment. The role of hsTnT in cardiovascular risk prediction in IA patients warrants further investigation.

## Supporting information

S1 File Stata file. Stata file containing all data underlying the findings described in the present study.
(DTA)
